# How Much Does it Cost to Expand a Protected Area System? Some Critical Determining Factors and Ranges of Costs for Queensland

**DOI:** 10.1371/journal.pone.0025447

**Published:** 2011-09-28

**Authors:** Vanessa M. Adams, Daniel B. Segan, Robert L. Pressey

**Affiliations:** 1 Australian Research Council Centre of Excellence for Coral Reef Studies, James Cook University, Townsville, Australia; 2 School of Biological Sciences, The University Of Queensland, St Lucia, Australia; Australian Wildlife Conservancy, Australia

## Abstract

Many governments have recently gone on record promising large-scale expansions of protected areas to meet global commitments such as the Convention on Biological Diversity. As systems of protected areas are expanded to be more comprehensive, they are more likely to be implemented if planners have realistic budget estimates so that appropriate funding can be requested. Estimating financial budgets *a priori* must acknowledge the inherent uncertainties and assumptions associated with key parameters, so planners should recognize these uncertainties by estimating ranges of potential costs. We explore the challenge of budgeting *a priori* for protected area expansion in the face of uncertainty, specifically considering the future expansion of protected areas in Queensland, Australia. The government has committed to adding ∼12 million ha to the reserve system, bringing the total area protected to 20 million ha by 2020. We used Marxan to estimate the costs of potential reserve designs with data on actual land value, market value, transaction costs, and land tenure. With scenarios, we explored three sources of budget variability: size of biodiversity objectives; subdivision of properties; and legal acquisition routes varying with tenure. Depending on the assumptions made, our budget estimates ranged from $214 million to $2.9 billion. Estimates were most sensitive to assumptions made about legal acquisition routes for leasehold land. Unexpected costs (costs encountered by planners when real-world costs deviate from assumed costs) responded non-linearly to inability to subdivide and percentage purchase of private land. A financially conservative approach - one that safeguards against large cost increases while allowing for potential financial windfalls - would involve less optimistic assumptions about acquisition and subdivision to allow Marxan to avoid expensive properties where possible while meeting conservation objectives. We demonstrate how a rigorous analysis can inform discussions about the expansion of systems of protected areas, including the identification of factors that influence budget variability.

## Introduction

International mandates such as the Convention on Biological Diversity (CBD) have become prominent in debates about extending protected areas [1]. Countries that sign the CBD commit to effectively protecting a portion of all their ecosystems. A recent study assessed progress towards the goal of protecting 10% of each ecoregion by 2010 and found that half of the world's ecoregions had not met this target [2]. Around the Conference of the Parties 2010, there have been notable political promises for expanded protected areas. For example, in 2008, the Democratic Republic of Congo announced it would double its protected area extent to 30 million ha [3]. More recently, the European Union promised to protect at least 20% of land by 2020, also doubling its current protected area estate [4]. Unfortunately there is often a gap between political promises and the actual funding available to achieve them. This could reflect the pervasive underfunding of conservation activities globally [5]. However, the shortfall in funding could also derive from the lack of comprehensive financial estimates of large-scale expansions of protected areas.

Constraints on funding for conservation have motivated global financial analyses to estimate the costs of conservation commitments [6], [7]. However, these have rarely been complemented by fine-scale estimates of the spatially variable conservation costs based on mapping of the ecosystems or other features that should be represented [8], [9]. Many planning studies have demonstrated that conservation objectives can be met more cheaply with data on spatially variable costs. However, these studies rely on many untested assumptions about factors influencing cost estimates. For example some studies apply global scale estimates to local scale problems [10], [11] or assume acquisition of only native vegetation within properties [10], [12]. To estimate the actual costs of expanding protected areas, planners must move beyond single estimates based on dubious assumptions. Several frameworks for considering uncertainty, both ecological and financial, have been proposed [13], [14] and the uncertainty of cost estimates associated with specific conservation actions has been assessed [15], [16]. However, there has been no systematic exploration of the uncertainties associated with estimating the cost of expanding protected areas.

**Figure 1:**
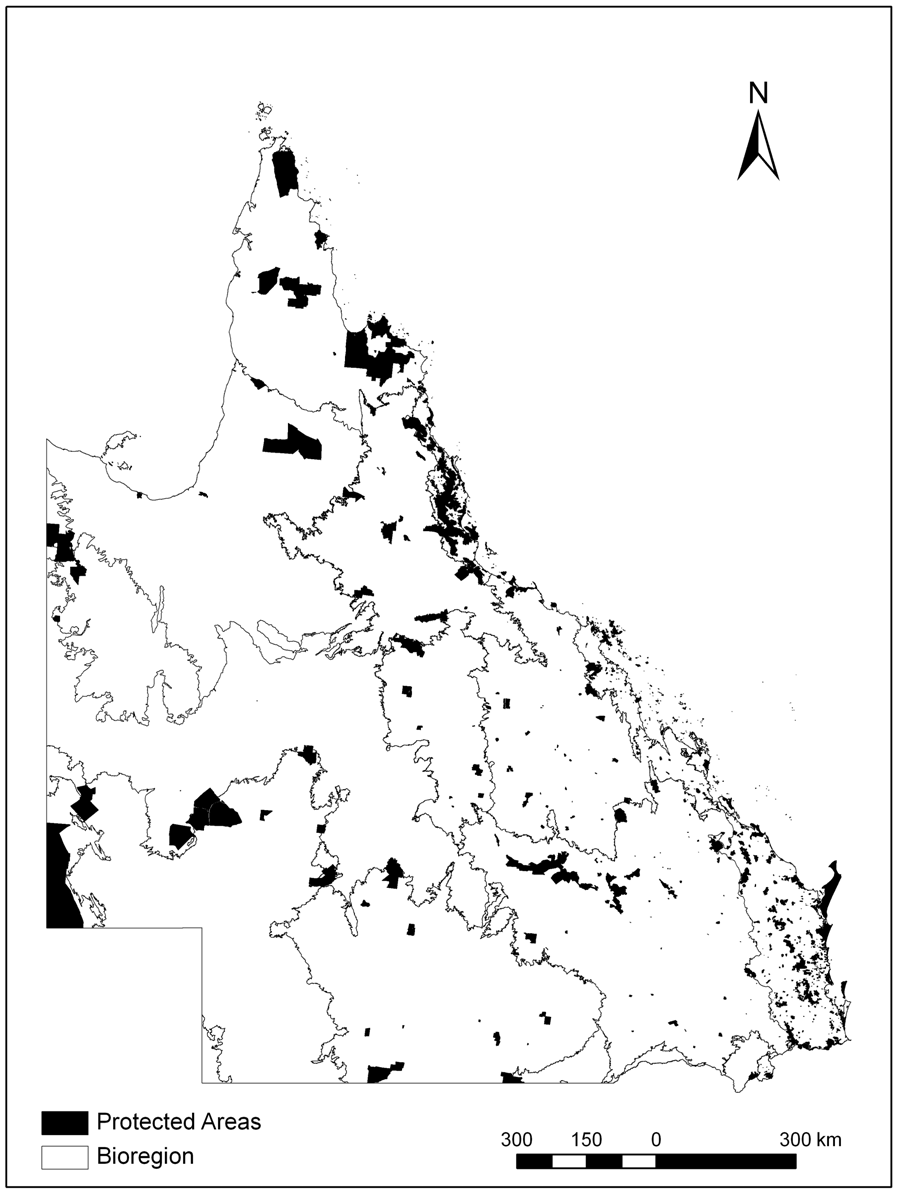
The state of Queensland, protected areas, and bioregions.

We identified ten factors likely to affect the cost of expanding protected areas (Table 1). Some of these relate to biological variables and are relatively well understood; but socio-political factors are also likely to be important determinants of costs. This study examines how three socio-political factors affect the expected cost of expanding protected areas in Queensland, Australia (Figure 1).

**Table 1:** Factors known or likely to affect the acquisition costs of protected areas.

Factor	Notes	References
Amount of biodiversity data	More complex data increase the total extent of conservation areas required to achieve conservation objectives because of imperfect spatial correlations between features.	[24], [27], [36], [45]*
Rarity and nestedness of species occurrences	Higher rarity of species (less spatial co-occurrence of species) increases the total extent of conservation areas required to represent them. Higher nestedness of species (more spatial co-occurrence of species) reduces the total extent of conservation areas required to represent them.	[45], [46]*
**Size of biodiversity objectives**	Larger conservation objectives for features such as species and vegetation types increase the total extent and total cost of conservation areas needed to achieve them.	[26], [27]* [47]
**Size of planning units (considered with landholder willingness to subdivide property)**	Smaller planning units require smaller total extents of conservation areas to achieve the same conservation objectives because they lead to less over-representation of objectives.	[25], [45]*
*Spatial variability in costs of planning units*	Efficiency gains of including costs in the planning process are strongly related to the relative variability of conservation costs.	[46]* [47], [48]
*Spatial correlation between biodiversity values and costs*	Efficiency gains of including costs in the planning process are strongly related to the correlation between conservation costs and benefits.	[47], [48], [49]
Connectivity of conservation areas	Grouping planning units so that they achieve objectives for connectivity (e.g. compactness, alignment to provide movement corridors) increases the total extent of conservation areas required to achieve other conservation objectives such as representation of species and vegetation types.	[50], [51]
Uncertainty about establishment costs of individual planning units	The actual establishment costs (e.g. opportunity or acquisition costs) of all planning units are seldom or never known with certainty, particularly across large regions. Typically, these costs must be estimated with surrogates (e.g. agricultural potential) or modeled from a limited number of data points (e.g. sales prices).	No studies have explicitly considered uncertainty of cost estimates, but several studies have developed frameworks for considering uncertainties [13], [14]
**Legal acquisition routes for protection of different tenures**	Depending on the tenure of land parcels, different legal routes are probably available for placing the parcel under protection (e.g. conservation easement or nature reserve programs for freehold land; stewardship requirements and payment programs for leasehold land). The total costs of achieving conservation objectives will vary strongly between different legal routes.	[22]
*Landholder willingness*	Landholders vary in their inclination to engage with conservation organizations. Issues include willingness to sell, willingness to negotiate portions of properties to be sold (i.e. willingness to subdivide property for sale), and willingness to participate in nature refuge or conservation management programs.	[22], [52]

The three factors considered directly in this study are shaded and bold. Factors in italics were considered indirectly through legal acquisition routes and subdivision of properties. Studies with asterisks estimated the effects on establishment costs as the number and/or total extent of conservation areas, which are likely to translate into effects on financial costs in all or most regions.

In 2008, the Queensland government promised to add ∼12 million ha to the network of protected areas, 4 million ha of which would be acquired for national parks, bringing the total estate to 20 million ha by 2020 [17]. There is flexibility in how this promise could be fulfilled, and different approaches will have different financial implications. The current 7.6 million ha of national parks were acquired over 100 years. The promised expansion therefore represents an unprecedented rate of addition of protected areas.

The primary objective of our study was to conduct a financial analysis of how much it will cost to expand the Queensland protected area estate, accounting for uncertainties around assumptions that make a range of potential costs more useful than a single figure. We therefore used a sensitivity analysis to examine two aspects of uncertainty in financial estimates: 1. the possible range of financial budgets required, depending on different assumptions made, termed “expected costs” and 2. the possible “unexpected costs” given departures from assumptions in the face of real-world constraints. We focused our study on three factors: the size of biodiversity objectives; willingness to subdivide properties; and legal acquisition routes dependent on tenure (Table 2). We selected these factors to reflect socio-economic assumptions that involve considerable uncertainty in the expansion of protected areas in Queensland.

**Table 2:** Factors (3) included in this study, associated variables (4) used in our calculations, and ranges of values to indicate uncertainties.

Factor	Variable considered	Range of uncertainties
Biodiversity objectives	Size of objectives for regional ecosystems	10%/1,000 ha or scaled objectives (2 values)
Landholder willingness to subdivide property	No subdivision/subdivision	No subdivision requires acquisition of entire property. Subdivision allows for acquisition of only remnant vegetation (2 values)
Legal acquisition routes for protection of different tenures	Freehold acquisition routes	0–40% of properties purchased (in 10% increments), with the remainder placed in Nature Refuge (5 values)
	Leasehold acquisition routes under the Delbessie Agreement	0–90% of properties purchased (in 10% increments), 5% of property leases under terminal lease renewal, with the remainder placed in Nature Refuge (10 values)

The full factorial design required 200 scenarios to consider all combinations of values (2×2×5×10).

We used sensitivity analysis because the promised rate of expansion of the protected area estate is unprecedented and not reflective of previous acquisitions. It was therefore not possible to derive accurate financial estimates from historic data. Historically, parks have not been located strategically, but rather in response to political imperatives, such as the Wet Tropics World Heritage Area, or based on ad hoc responses to availability of land and lengthy negotiations with landholders. Furthermore, future expansion of protected areas in Queensland will involve recent legal initiatives with uncertain applications. One of these initiatives is the Nature Refuge program which allows landholders to voluntarily place portions of their properties under conservation covenants. The covenants are attached to land titles in perpetuity, stipulate nature conservation as the primary use and constitute IUCN category VI protected areas. Nature refuges therefore contribute to the national reserve system and to meeting global commitments such as the CBD. However, NatureAssist, which involves competitive bidding by landholders to support management of Nature Refuges, provided its first round of funding only in 2007. It is unclear whether the last 5 years of funding will reflect future funding, or whether this will be sufficient incentive to engage many more landholders. A second recent legal initiative is the Delbessie Agreement, legislated in the last 5 years and untested in implementation. The Delbessie Agreement is a framework of legislation, policies and guidelines supporting the environmentally sustainable, productive use of rural leasehold land for [18]. The lessees with properties identified as having conservation value can enter into a Nature Refuge agreement and be rewarded with a 10-year lease extension. Alternatively, they can elect to have their properties acquired. The percentage of lessees choosing either option is very difficult to estimate. Given the Government's commitment to a rapid expansion of protected areas in Queensland and these two relatively untried legal instruments, we set out to explore the effects on costs of uncertainties around several influential variables.

## Results

For each of our 200 scenarios we used Marxan [19] to select properties to meet our conservation objectives and calculated the total area selected and total expected costs. We then calculated the potential unexpected costs for the areas selected in each scenario given certain deviations from assumptions. The total additional area of land required to achieve objectives was much larger than the government's promised 12 million ha. The minimum area added to the reserve system across all scenarios was 18 million ha and the maximum was 29 million ha with an average of 23 million ha. In all scenarios, the full extent of available State land (∼2.3 million ha) was selected by Marxan due to its low cost compared to other tenures. Total costs ranged from $214 million to $2.9 billion, with larger costs associated with larger percentage acquisition assumptions.

We identified the scenarios most likely to reflect the government's commitment to acquire 4 million additional ha of national park, which we also interpreted as 33% of the total expanded area required to meet objectives. Given an average of 23 million ha of additional protection needed, adding only 4 million ha to the national park estate would mean that covenants on private land would be relied upon heavily to meet conservation objectives. On the assumption that the government will commit to acquire 33% of the required land to meet conservation objectives, the area needing acquisition actually ranged from about 6 to 10 million ha. The total expected costs of scenarios involving an additional 4 million ha or 33% acquisition for national parks ranged from $250 million to $1.6 billion. The minimum cost estimate of $250 million was based on the 10%/1000 ha objectives and assumed property subdivision, 30% freehold acquisition, and 0% leasehold acquisition. However, given the uncertainties about implementation of the Delbessie Agreement and the potential need to purchase larger amounts of leasehold land, the maximum expected cost could rise to $2.3 billion (based on the scaled objectives with no subdivision, assuming 0% freehold acquisition and 90% leasehold acquisition).

**Figure 2:**
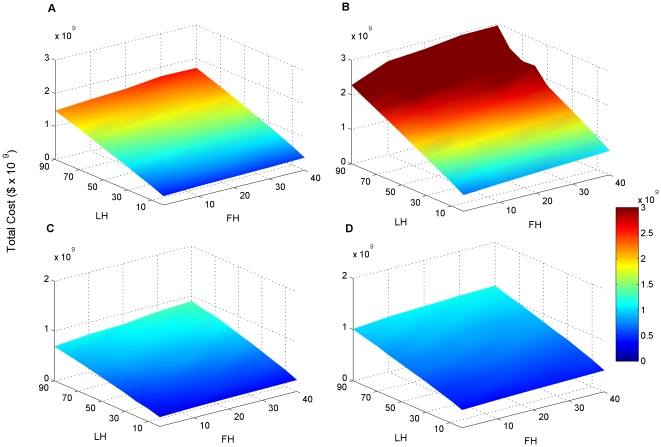
Total cost (billions of Australian dollars) as a function of variable percentages of leasehold and freehold land purchased. Expected total costs are plotted on the z-axes, percentages of leasehold (LH) purchased are on the y-axes, and percentages of freehold (FH) purchased are on the x-axes. A) 10%/1,000 ha objectives and no subdivision of properties; B) scaled objectives and no subdivision of properties; C) 10%/1,000 ha objectives and subdivision of properties; D) scaled objectives and subdivision of properties.

Total expected costs were 50–80% larger for scaled objectives which required ∼17% more area (Figure 2). For both sets of objectives, the expected costs of subdivision scenarios were 5–30% lower than those without subdivision (Figure 2). Biodiversity objectives interacted with percentage acquisition assumptions to influence expected costs. Expected costs responded linearly to increasing percentages of both freehold and leasehold land purchased for both sets of objectives (Figure 2). In the multiple regression of total cost against all factors, objectives and subdivision had the largest effects (Table 3). On average, scaled objectives were about $489 million more expensive than 10%/1,000 ha objectives. Subdivision of properties for purchase of only remnant vegetation reduced total costs on average by $225 million. Percentage purchase of leasehold land had almost three times the effect on total cost as percentage purchase of freehold land.

**Table 3:** Multiple regression model of total cost.

		Independent Variables (coefficient, *t*)				
	Intercept	pct_LH_ a	pct_FH_ b	Subdivision	Scaled objectives	Overall R^2^
Total cost	242 124 199, 104.13	1 538 538 297, 518.9	455 236 918, 75.60	−225 607 405, −132.46	489 342 331, 287.3	0.949

All variables are highly significant (p<0.001). Coefficients represent the dollar change in total cost.

a pct_LH_ Percent leasehold assumption was expressed in proportional form (i.e. 10% coded as 0.10). Therefore, the coefficient indicates that a 10% increase in leasehold purchase gives a dollar change in cost of 1 538 538 297×0.1 or 153 835 829.

b pct_FH_ Percent freehold assumption was expressed in proportional form (i.e. 10% coded as 0.10). Therefore, the coefficient indicates that a 10% increase in freehold purchase gives a dollar change in total cost of 455 236 918×0.1 or 45 523 691.

**Figure 3:**
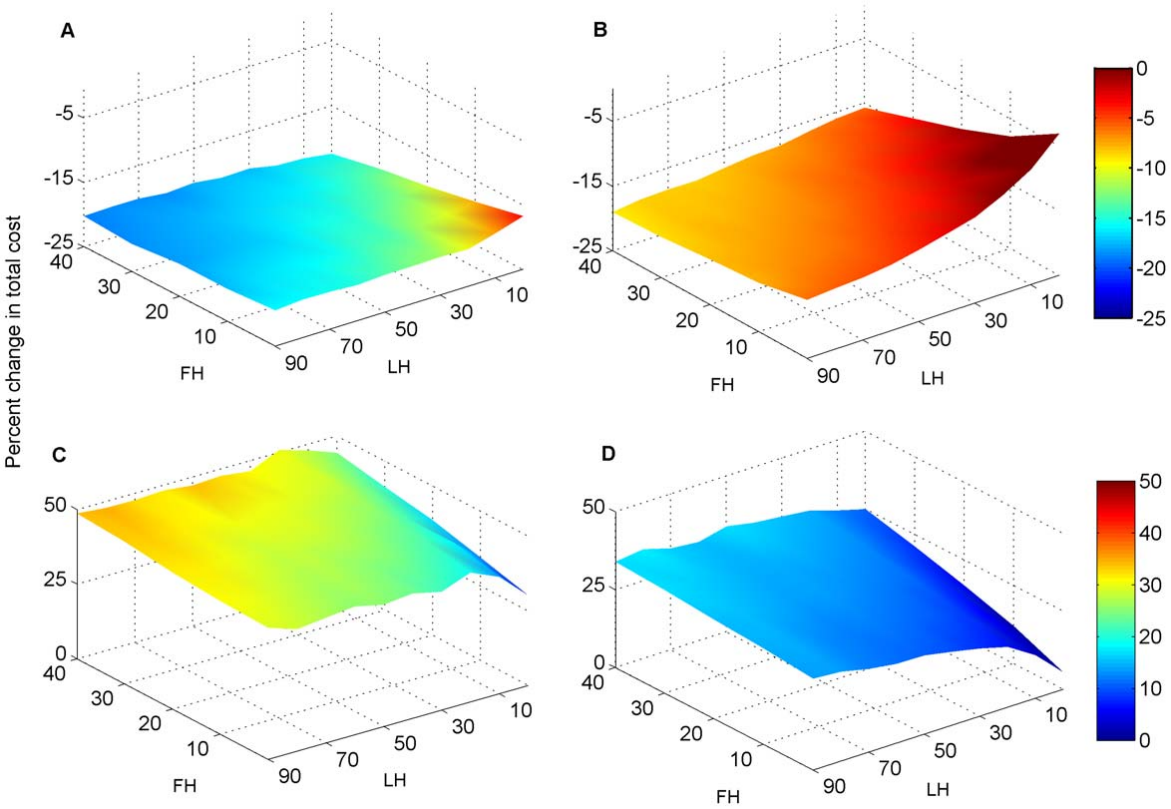
Percentage change in total cost due to unexpected subdivision conditions as a function of variable percentages of leasehold and freehold land purchased. Percentage deviations from expected total costs are plotted on the z-axes, percentage purchases of leasehold (LH) are on the y-axes, and percentage purchases of freehold (FH) are on the x-axes. A) Percentage reduction in cost if all properties can be unexpectedly subdivided under the 10%/1,000 ha objectives and no subdivision assumption; B) Percentage reduction in cost if all properties can be unexpectedly subdivided under the scaled objectives and no subdivision assumption; C) Percentage increase in cost if all properties are unexpectedly impossible to subdivide under the 10%/1,000 ha objectives and subdivision assumption; D) Percentage increase in cost if all properties are unexpectedly impossible to subdivide under the scaled objectives and subdivision assumption.

Predictably, unexpected ability to subdivide properties reduced costs and unexpected inability to subdivide properties increased costs, but the responses were non-linear across scenarios (Figure 3). The largest reductions in costs when assumed inability to subdivide proved incorrect were for 40% freehold purchase and 0% leasehold purchase for the 10%/1,000 ha objectives, and 40% freehold purchase and 90% leasehold purchase for the scaled objectives (Figure 3a,b). Similar combinations of purchases produced the largest increases in costs when assumed subdivision was not possible. The largest increases occurred for 40% freehold purchase and 20% leasehold purchase for the 10%/1,000 ha objectives, and 40% freehold purchase and 80% leasehold purchase for the scaled objectives (Figure 3c,d).

The sensitivity of total costs to unexpected 10% increases in purchases of leasehold land increased with larger expected percentage purchases of freehold land and decreased with larger expected percentage purchases of leasehold land (Table 4). However, the sensitivity was larger for the scaled objectives, with a maximum increase of $279 million compared to $217 million for the 10%/1,000 ha objectives. The opposite trend applied to unexpected 10% increases in purchase of freehold land. Sensitivity decreased with larger expected percentage purchases of freehold land and increased with larger expected percentage purchases of leasehold land (Table 5). Average increases in costs were higher for the 10%/1,000 ha objectives for 0% freehold purchase but, for all other freehold percentage purchase scenarios, average increases in cost were higher for the scaled objectives.

**Table 4:** Sensitivity to 10% change in expected purchase of leasehold land for both objectives (10%/1,000 ha on left and Scaled on right), holding all other assumptions constant.

(a)											
	10%/1,000 ha objectives						Scaled objectives				
LH, FH	0	10	20	30	40	LH, FH	0	10	20	30	40
0	186	210	216	218	217	0	249	266	276	279	279
10	152	163	172	175	177	10	212	223	228	234	233
20	141	147	155	159	162	20	202	209	213	219	219
30	138	142	146	152	155	30	201	206	210	212	217
40	135	138	142	147	150	40	198	202	206	209	210
50	134	137	140	143	145	50	199	202	205	208	210
60	133	135	138	140	144	60	197	199	202	205	241
70	133	135	136	139	142	70	196	199	201	203	206
80	132	133	135	135	138	80	195	198	200	202	204
90	130	130	133	143	136	90	196	231	233	242	237

(b)											
	10%/1,000 ha objectives						Scaled objectives				
LH, FH	0	10	20	30	40	LH, FH	0	10	20	30	40
0	133	153	156	159	160	0	179	193	197	204	204
10	116	124	142	133	137	10	160	167	184	175	178
20	109	113	119	122	126	20	158	163	165	170	172
30	108	110	114	119	121	30	155	160	162	165	168
40	105	107	110	113	116	40	155	159	160	163	165
50	105	107	109	123	113	50	155	157	160	174	167
60	103	104	106	107	110	60	153	155	157	158	159
70	103	103	105	107	108	70	152	154	155	157	158
80	101	102	103	105	106	80	153	154	156	157	185
90	102	102	103	105	106	90	152	153	155	156	157

Sensitivity is expressed as the increase (AUD$ million) in total cost for an unexpected 10% increase in purchase of leasehold. The expected percentages of leasehold (LH) and freehold (FH) purchase that serve as baselines for the increases are given as rows and columns, respectively. For example, dollar values in the row corresponding to 20% leasehold correspond to an unexpected need to purchase 30% of leasehold land. No changes in expected purchases of freehold land apply here. (a) assuming no subdivision of properties; (b) assuming subdivision and purchase only of remnant vegetation.

**Table 5:** Sensitivity to 10% change in expected purchase of freehold land for both objectives (10%/1,000 ha on left and Scaled on right), holding all other assumptions constant.

(a)											
	10%/1,000 ha objectives						Scaled objectives				
LH, FH	0	10	20	30	40	LH, FH	0	10	20	30	40
0	137	23	19	19	21	0	117	58	54	51	51
10	142	38	25	22	23	10	139	67	59	56	56
20	157	44	30	25	26	20	141	72	66	58	58
30	156	51	39	30	27	30	147	79	68	65	59
40	158	53	42	33	30	40	148	84	71	66	64
50	154	57	45	38	35	50	149	89	75	68	63
60	156	60	46	41	36	60	150	89	77	70	66
70	157	57	50	42	37	70	152	88	81	74	67
80	161	63	48	47	42	80	150	89	82	76	71
90	161	64	51	39	44	90	146	92	82	71	70

(b)											
	10%/1,000 ha objectives						Scaled objectives				
LH, FH	0	10	20	30	40	LH, FH	0	10	20	30	40
0	88	14	12	11	11	0	79	34	30	28	27
10	106	24	18	12	12	10	91	41	39	32	30
20	103	32	19	16	14	20	96	45	39	33	32
30	113	36	26	17	16	30	96	46	42	37	33
40	116	37	27	23	19	40	92	49	44	39	36
50	111	38	31	27	22	50	98	53	43	43	37
60	115	39	31	28	25	60	106	54	46	42	40
70	114	43	34	28	27	70	99	55	48	42	41
80	107	42	35	29	30	80	101	58	48	45	39
90	107	43	38	32	29	90	106	60	50	46	43

Sensitivity is expressed as the increase (AUD$ million) in total cost for a 10% increase in purchase of freehold. The expected percentages of leasehold (LH) and freehold (FH) purchase that serve as baselines for the increases are given as rows and columns, respectively. So, for example, dollar values in the column corresponding to 20% freehold correspond to an unexpected need to purchase 30% of freehold land. No changes in expected purchases of leasehold land apply here. (a) assuming no subdivision of properties; (b) assuming subdivision and purchase only of remnant vegetation.

## Discussion

While governments commonly identify lofty multi-year conservation goals, it is uncommon for them to estimate the required costs. When financial estimates are released, they might be serious underestimates, reflecting the pervasive problem of under-funding conservation. The Queensland government estimated the cost of its commitment of 20 million ha of protected areas by 2020 at $120 million [17]. Recognizing that our cost estimates include increases in funding for management while the government estimate was strictly for acquisition, the figure of $120 million is still well below our expected cost range (Figure 2). Our lowest total estimate was $214 million and required several extremely optimistic assumptions, including that all land would be acquired through Nature Refuges, and that remnant vegetation would always be subdivided out of properties to more efficiently achieve objectives. In the range of scenarios that best matched the political promise by the Queensland government to acquire 4 million ha or 33% of the expanded area, the minimum cost estimate was $250 million.

### Cost variability

Total costs across our 200 scenarios varied by an order of magnitude from $214 million to $2.9 billion. All of the factors tested influenced this variation, with objectives and subdivision the most important. Cost was linearly influenced by percentage leasehold acquisition, which is to be expected because of the linear increments in percentage of properties acquired. However, when we considered unexpected costs, they responded non-linearly to unexpected inability to subdivide and percentage purchase of freehold. This is because freehold properties are small, much more expensive per ha than leasehold properties and have smaller proportions of native vegetation than leasehold properties. The wide range of cost estimates, the influence of underlying assumptions, interactions between assumptions, and non-linear responses make *a priori* estimates and general rules of thumb difficult to derive.

Many of the effects of key factors are difficult to anticipate without the kinds of analyses presented here. For example, scaled objectives raised costs by 50–80% despite the targeted area being about 2 million ha smaller. There were two reasons. First, rarer regional ecosystems had larger proportional objectives with the scaled method and these ecosystems were more expensive to protect per unit area. The Pearson correlation between log(regional ecosystem area) and log(regional ecosystem average cost per ha) was −0.117 (p<0.001). The second reason was that larger scaled objectives reduced spatial flexibility for representing rarer regional ecosystems, providing few or no alternatives to more expensive properties. The costs of conservation can therefore be more sensitive to objectives for individual biodiversity features than to aggregate area goals such as percentages of a state.

### Dealing with uncertainty about costs

The amplitude of unexpected increases and reductions in costs could help to guide what assumptions are the most conservative. We define financially conservative assumptions here as those that safeguard against large cost increases while allowing for potential financial windfalls. We measured uncertainty by estimating how expected costs were changed when assumptions did not hold true. The largest deviations from expected costs occurred when very low acquisition assumptions (∼0–20%) proved inaccurate. When acquisition assumptions were low, property values were effectively smoothed to obscure spatial variability in purchase costs. This can result in selection of expensive properties and expose planners to large unexpected costs if landholders are less receptive to Nature Refuges than assumed. Selections of properties under larger acquisition assumptions recognized variability in costs and avoided expensive properties, where there were choices, so unexpected costs represented smaller increases when assumptions did not hold (Table 5). Therefore, a conservative approach would use larger acquisition assumptions to allow the selection algorithm to avoid expensive properties where possible.

Unexpected costs or savings resulting from inaccurate subdivision assumptions indicated that potential costs far outweighed potential savings. When subdivision unexpectedly occurred after selection of areas, costs were reduced by only ∼15%. In contrast, when subdivision unexpectedly did not occur, costs increased by ∼45%. A conservative approach – assuming no subdivision - therefore resulted in only a small loss of efficiency but avoided a large financial risk. Unexpected increases or reductions in costs related to subdivision also interacted with assumptions about percentages of freehold and leasehold land that would be acquired (Figure 3), so potential increases were up to 50% in our analyses.

### Lessons for Queensland

Expanding the Queensland protected area system could incur a wide range of financial costs, depending on biodiversity objectives and socio-political conditions encountered. While this variation might be reduced by estimating plausible bounds for key factors, a single exact estimate cannot be provided because there will always be uncertainty associated with *a priori* assumptions. The narrowed range of scenarios based on the acquisition promise of 4 million ha or 33% still resulted in costs varying by an order of magnitude due to unavoidable uncertainty around the responses of leaseholders to the Delbessie Agreement. Because the Queensland government is required under this Agreement to immediately purchase leasehold properties with conservation value, we explored the full range of leasehold percentage purchases, resulting in a wide cost range. Even long experience with acquisition of individual properties, sometimes involving protracted negotiations, does not necessarily equip an agency to accurately cost a massive expansion of protected areas across many hundreds of properties, involving many hundreds of landholders who have not previously dealt with agency officers, while implementing new, and largely untested, legislation.

To avoid undesirable surprises in Queensland, planners should not assume that large percentages of land will be protected through Nature Refuges at low cost to the government. Similarly, they should use conservative assumptions about subdivision. Assumptions of larger percentages purchased and smaller proportions of properties subdivided will cause the selection algorithm (in our case, Marxan) to avoid expensive properties with extensive clearing of native vegetation. While increasing expected budgets, this would avoid large unexpected increases in costs. The selected properties could then be analyzed in detail with respect to remnant vegetation and attitudes of landholders to narrow the range of expected costs, targeting specific properties for subdivision and acquisition routes.

Narrowing the range of potential costs of an expanded protected area system in Queensland requires a better understanding of landholders' interests in selling their properties in whole or part, negotiating leases, or participating in the Nature Refuge program, and how these interests vary geographically and by land use. Likely levels of participation in conservation programs as well as potential costs can be determined through local-scale experiments such as tendering processes or closed-bid auctions [20], [21]. This in-depth analysis would, however, be very difficult and costly across Queensland. Reducing variation in financial estimates might also be helped by decision rules such as only considering properties with at least 50% remnant native vegetation [22].

Particular care should also be given to setting interim versus long-term objectives, especially for the small regional ecosystems that can influence costs and for any regional ecosystems threatened by further reductions in extent. Achieving longer-term, scientifically defensible targets, which might have high financial costs, in the context of continuing, incremental depletion of native vegetation, requires an explicit strategy for scheduling conservation actions [23], not evident in current Queensland policy.

### General conclusions

Although our analysis focused on Queensland, the same factors are likely to affect the cost of conservation in other parts of the world. Our findings are generic, in that the estimated costs of expanding protected area systems to meet policy goals or political commitments are likely to vary widely and to be highly sensitive to assumptions about influential factors.

In our study, subdivision and conservation objectives were particularly important in influencing the costs of conservation for very different reasons. The ability to subdivide properties dramatically affected the amount of land required to meet the conservation objectives. This result is general and not context-specific and has been noted previously in relation to the total extent of selected areas [24], [25]. Conservation planners should be aware of the sensitivity in their spatial selections and subsequent financial estimates when assuming that landholders will be willing to subdivide properties. Conservation objectives affected the amount of land acquired, the total cost of achieving conservation objectives, and the spatial flexibility in achieving those objectives. The spatial options available to meet objectives are context-specific, but other studies have found effects of conservation objectives on total area and costs [26], [27].

Comparisons of the potential political pathways for acquiring and protecting land have been largely neglected in the literature. However, with the recent increase in conservation programs on private lands [21], [28], [29], the cost differences between the traditional acquisition route and alternative approaches to protection are likely to become more apparent. This means that, when budgeting for conservation, assumptions regarding how lands are protected will become increasingly important. The factors we selected provide important insights into the types of impacts that assumptions can have on estimated financial costs. However, conservation planners should evaluate the context-specific assumptions they make based on the potential of those assumptions to change conservation requirements. This means that factors not discussed here might be important in other studies.

While conservation planners cannot be expected to have infallible foresight into the accuracies of their assumptions, they can be expected to be transparent about their uncertainties [30]. Given the global under-funding of conservation [31] and recent commitments to extensive, but uncosted, expansions of protected area systems, planners must be forthright in providing realistic estimates and avoiding underestimates that could compromise both their credibility and conservation outcomes.

## Materials and Methods

### Planning region

The study region included the whole state of Queensland, Australia, with a total area of 185 million ha of which approximately 8 million ha is protected (Figure 1). We implemented our sensitivity analysis by estimating the total financial cost of the protected area expansion for incremental changes in four variables, associated with our three key factors (Table 2). We selected the four variables and appropriate values for them based on expert interviews conducted with the Queensland Department of Environment and Resource Management (DERM). We then used Marxan [19], a reserve design tool, to estimate the total financial costs for each scenario (see Reserve Design section). Marxan is a commonly used conservation planning software that selects sets of areas, in this case properties, to meet conservation objectives at a minimum “cost”. The objective function can include costs of areas, total boundary length of areas, and penalties for failing to meet conservation objectives. For each scenario, we used Marxan's best run (the run with the smallest objective function) and calculated the total area selected and total expected costs. To ensure that we explored ranges of each variable while holding all other variables constant, we used a full factorial design resulting in 200 reservation scenarios (Table 2). Details on methods for each of the variables are below.

### Biodiversity objectives

Based on conversations with DERM we developed two different methods for setting conservation objectives that reflected the department's thinking and minimum policy requirements for the Commonwealth Government to achieve 10% protection of all bioregions. We do not endorse these objectives as ecologically adequate, but present them here to illustrate their effect on costs of protected areas.

At the core of Australia's biodiversity conservation strategy are the goals for protected areas to be comprehensive, adequate, and representative. Comprehensiveness refers to the need to sample all bioregions; adequacy relates to the persistence of biodiversity; and representativeness indicates how well ecosystems are sampled within bioregions [32]. To address both comprehensiveness and representativeness, we used the Queensland Herbarium's regional ecosystem mapping of remnant vegetation at 1∶50,000 to identify our conservation features [33]. Regional ecosystem mapping is the most comprehensive, fine-scale data on vegetation formations available for Queensland. Classification of regional ecosystems considers bioregional boundaries, vegetation structure, geology, landform, and soil [34], [35]. There are thirteen bioregions (Figure 1) and over 1300 regional ecosystems mapped across Queensland. We used the estimated pre-clearing extent of each regional ecosystem for setting biodiversity objectives [36], although our selections of new areas were based on remnant native vegetation.

The first method for defining objectives (“10%/1,000 ha”) was derived from minimal policy requirements [1], [37]. We used a base objective of 10% of the estimated pre-clearing extent of each regional ecosystem. If this percentage was less than 1,000 ha, we set the objective to 1,000 ha. If the pre-clearing extent was less than 1,000 ha, we set the objective to the pre-clearing extent (i.e. 100%). We then expressed these objectives as ha of remnant vegetation. If the remnant area of a regional ecosystem was smaller than its objective, we trimmed the objective to the remnant area.

The second method (“scaled objectives”) used a power function to scale objectives based on extent of vegetation. This method reflects the fact that a very extensive ecosystem might not need 10% protection to ensure long-term viability, whereas a small, heavily cleared ecosystem could need a much larger percentage protected. If the pre-clearing extent was less than 1,000 ha, the objective was set to pre-clearing extent (i.e. 100%). If the pre-clearing extent was 1,000 ha or larger, we used the following equation with a power value of *p* = 0.5:
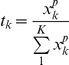
where *t_k_* is the objective for regional ecosystem *k*, expressed as a proportion, *p* is the power, and *x* is the pre-clearing extent of regional ecosystem *k*. More extensive regional ecosystems (representing larger proportions of total pre-clearing vegetation) therefore had larger objectives, but the power function produced a diminishing rate of increase in objectives with increasing pre-clearing extent. We multiplied each objective by pre-clearing extent to express it in ha and, if necessary, trimmed it to total remnant area.

**Figure 4:**
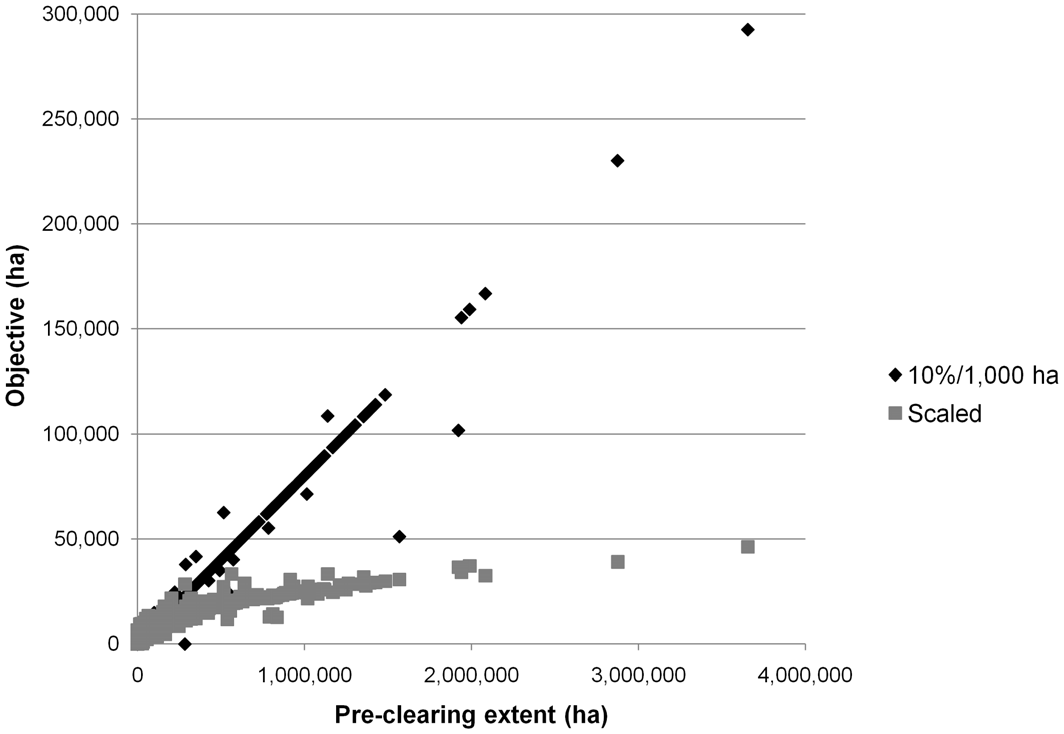
Objectives for regional ecosystems plotted against estimated pre-clearing extents for both methods.

Objectives for the 10%/1,000 ha method totaled about 12.5 million ha compared to about 10.6 million ha for scaled objectives. However, the methods differed more importantly in their objectives for individual regional ecosystems. Compared to the 10%/1,000 ha objectives, scaled objectives gave larger values to regional ecosystems with smaller pre-clearing extents and smaller values to regional ecosystems with larger pre-clearing extents (Figure 4).

### Subdivision of properties

Willingness of landholders to subdivide properties for sale is likely to be highly variable and difficult to estimate *a priori* for large numbers of properties managed by people with no previous contact with DERM. Acquisition of whole properties is more expensive and will often involve purchase of land without native vegetation. Acquisition only of native vegetation is less expensive and more targeted. To bound our estimates of costs, we compared two subdivision scenarios, one without subdivision and one involving subdivision to protect only remnant native vegetation. In scenarios with subdivision, we assumed homogeneous cost across each property (below) and pro-rated the cost for remnant vegetation. In places, our subdivision assumption will underestimate costs, with protection of only unrealistically small patches of native vegetation. However, it reflects assumptions made in the literature [38], [39].

### Legal acquisition routes

We explicitly considered the multiple acquisition routes for freehold (privately owned) land (Table 6). Freehold land can be purchased by the state at market value. Alternatively, the owner can retain the property with a voluntary Nature Refuge covenant on all or part of it, with no acquisition cost. The two acquisition routes for freehold land mean that estimates of establishment costs are uncertain, depending on assumptions about willingness of landholders to sell or negotiate Nature Refuges. Additionally, the Queensland Government's commitment to an eight-fold increase in participation in the Nature Refuge program might not reflect the actual willingness of landholders. Based on the relative cost of freehold, leasehold and state land, we assumed that the government would purchase at most 40% of freehold land at market price and the remainder would be negotiated as Nature Refuges (Table 2). This reflects the fact that freehold land can be very expensive and many freehold properties are unsuitable as national parks because they are small and heavily cleared with only fragments of native vegetation remaining. This reasoning reflects DERMs approach, so our range of acquisition assumptions (0–40%) likely reflects the scenarios considered by government.

**Table 6:** Pathways of land into the Queensland protected area system and associated costs in relation to tenure.

Pathway into protected area system	Cost of acquiring	Transaction cost	Annual management costs
Freehold voluntary purchase to create new park	Market value	$20,000 per sale for coastal properties, $15,000 elsewhere	$8.12 per ha
Leasehold voluntary purchase to create new park	Market value	$20,000 per sale for coastal properties, $15,000 elsewhere	As above
Leasehold Future Conservation Area (FCA): terminal 30-year lease with transfer to parks system at expiry	Value of improvements (difference between market and unimproved land value)	$20,000 per sale for coastal properties, $15,000 elsewhere	As above
State Forests or other State land transfer to parks system	None	None	As above
Freehold converted to Nature Refuge by covenant	None	$20,000 per sale for coastal properties, $15,000 elsewhere	$3.82 per ha
Leasehold converted to Nature Refuge by covenant	None	$20,000 per sale for coastal properties, $15,000 elsewhere	As above

We also considered the multiple acquisition routes for leasehold land (land owned by the State and leased for set periods for agricultural or grazing) (Table 6). Under the Delbessie Agreement [18], landholders with leases currently up for renewal identified as conservation priorities have three options: immediate sale at market value; 30-year terminal lease with payments for improvements at the end of this term; and renewal of lease with a covenanted Nature Refuge on part of the property. If the landholder elects to sell immediately, the Queensland government is required to purchase the property at market value. While conversations with DERM indicate that 30% of leaseholders are expected to sell, with the remainder negotiating renewed leases with Nature Refuges, this assumption is untested. Little is known about leaseholders' responses to the Delbessie Agreement, so the establishment costs associated with leasehold purchases and renewals are highly uncertain. We therefore explored acquisition levels up to 90% at market price, with the remainder negotiated as Nature Refuges (Table 2).

For each level of assumed acquisition of freehold and leasehold properties, we calculated the expected cost of properties as: x*costNR+y*costpurchase, where *x* and *y* are the percentages assumed for Nature Refuge and acquisition, respectively, and *costNR* and *costpurchase* are the costs of Nature Refuge and acquisition, respectively. For both freehold and leasehold land, we avoided *a priori* allocation of properties to individual acquisition routes to avoid idiosyncratic correlations between cost and particular examples of regional ecosystems.

### Planning units and conservation costs

Legal properties are the units with which managers implement conservation actions, but previous analyses of conservation costs have rarely used cadastral boundaries. To more accurately estimate the costs and extent of land needed to meet objectives, we used legal property boundaries to define planning units [40]. Our subdivision scenarios involved selection of properties but costing only of native vegetation.

For each property, we used data on tenure, unimproved land value, and sales prices [40], [41], [42] to estimate the cost of acquisition, considering all acquisition routes (Table 6). Dates of land valuation and sales data varied, so we adjusted all values to 2008 dollars using published annual interest rates [43]. We estimated market values from recent sales of properties in Queensland from 2000–2008 with hedonic modeling [44]. We considered a standard ordinary least squares (OLS) model as well as a geographically weighted regression model (for comparison of results see Information S1 for details). We tested the OLS residuals for spatial autocorrelation using the Moran I Statistic, which rejected the null hypothesis that there was no spatial autocorrelation (p<0.01). We therefore modeled sales value with geographically weighted regression in ArcGIS 9.3, which analyses spatially variable relationships between the dependent and independent variables (for full details of variables see Information S1).

For our geographically weighted regression, we first considered the entire state. However, because coastal properties in Queensland have different characteristics to those elsewhere (for example, average size of coastal properties is 1/40 that of others), analyzing the entire state led to local multi-collinearities in coastal properties. We therefore applied geographically weighted analysis for coastal properties and the remainder of properties separately. For coastal properties, the only predictor without strong local correlations was *log(cleared area, ha)*. The local R^2^ for coastal properties was lower than for others because of the lack of predictors available to capture potential for coastal development (adjusted R^2^ = 0.688). For the remainder of properties, predictors were *log(land value per ha)*, *log(cleared area, ha)*, *log(soil, ha)* and *log(distance to nearest town, km)* (adjusted R^2^ = 0.904).

**Figure 5:**
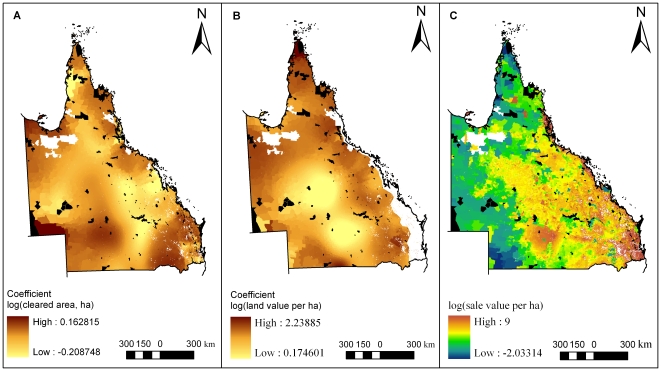
Maps of spatially variable coefficients based on geographically weighted regression conducted for coastal and non-coastal regions of Queensland. Current protected areas are shown in black for reference. White areas in the north-west have no vegetation mapping. A) Coefficient of log(cleared area, ha) from the geographically weighted regression for coastal and non-coastal regions. White areas in the south-east are properties without remnant vegetation and are excluded from our analysis. B) Coefficient of log(land value per ha) from the geographically weighted regression for non-coastal regions. The additional, continuous white area along the eastern seaboard is coastal Queensland, excluded from this model. North of this excluded region is Cape York Peninsula, considered separately in the model. C) Predicted log(sale value per ha) for properties with remnant vegetation. White areas in the south-east are properties without remnant vegetation and are excluded from our analysis.

The spatially variable coefficients for *log(cleared area, ha)* and *log(land value per ha)* and the final predicted sales values are in Figure 5. The coefficient for *log(cleared area, ha)* is of interest because this was the only predictor used across the entire state (including coastal areas). The coefficient for *log(land value per ha)* is of interest because land value is typically the only type of cost data used to estimate acquisition costs in other academic studies for Australia [38], [39]. The final sales values are easily interpreted for the state. Noticeable low-cost regions along the coast corresponded to defense properties. High-cost inland properties followed the major inland highway and clustered around agricultural and mining towns.

For each property in each scenario, we calculated the total cost (*TC*) as an expected value based on the respective percentage acquisition assumptions, using the following equation:
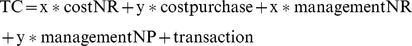
where *x* and *y* were the percentages assumed for Nature Refuge and acquisition, respectively, *costNR* and *costpurchase* were the costs of Nature Refuge and acquisition, respectively, *managementNR* and *managementNP* were the one-year increase in management costs for Nature Refuge and national park, respectively, and *transaction* was the transaction cost (Table 3). Transaction costs were based on estimates by DERM. Management costs were based on gross hectares added, current average annual management costs of $8.12 per ha of national park (from the most recent available financial expenditures at the time of analysis, 2006–7), and average annual NatureAssist cost for Nature Refuges of $3.82 per ha (from the most recent available financial expenditures at the time of analysis, 2007). For properties in the expanded protected area system, we considered management costs for a single year with no discounting.

### Reserve design

We accounted for the contribution to objectives of existing protected areas by locking them into the solutions in all scenarios. We used Marxan to design additional reserves that met the remaining portions of all objectives while minimizing total costs [19]. For each of our 200 reservation scenarios, we ran Marxan with 100 repeat runs and no configuration constraints (boundary length modifier or BLM set to 0). Spatial design criteria should reflect differences in regional ecosystems, population density, and management objectives, so applying universal criteria across the entire state of Queensland would have been inappropriate.

### Analysis of reserve design solutions

We compared the scenarios by recording for each, from the ‘best’ solution (with the smallest objective function across 100 repeat runs), the total extent of selected areas, excluding existing reserves, and their total cost.

We used a two-step process to assess the sensitivity of cost estimates to values of our variables. First, we calculated the total cost that would apply if all assumed values of key variables held true (“expected cost”). Expected costs would apply, for example, if areas were selected on the assumption that no subdivision of properties would be possible and, after negotiation with landholders, this proved to be the case. We recorded the expected cost for each run and regressed expected cost against our four variables: percentage acquisition route for freehold land (percentage, 0–40), percentage acquisition route for leasehold land (percentage, 0–90), type of objective (binary, 10%/1,000 ha or scaled), and subdivision (binary, yes or no). Second, we calculated the cost of each scenario with different amounts of deviation from scenario assumptions (“unexpected costs”). For example, areas might be selected and total costs estimated on the assumption that 50% of leasehold properties would be purchased. If the percentage requiring purchase was actually 60%, then 10% of properties expected to be inexpensive would prove to be otherwise and unexpected costs would apply. The increase in total cost would be greater than if 60% acquisition had been expected initially, which would have shifted selections more towards properties with lower acquisition costs. We calculated both the absolute and percentage differences between expected and unexpected costs.

This approach to estimating sensitivity to key variables is important because *a priori* assumptions about values of variables, no matter how well informed, will always be inaccurate to some extent. Our approach “commits” the agency to configurations of new reserves based on assumptions holding, even if the assumptions prove incorrect. In reality, the agency would revise configurations accordingly, although it might not be able to revise its financial estimates so easily. Nonetheless, our approach indicates how inaccurate cost estimates can be if values of key factors deviate from expected.

## Supporting Information

Information S1 **Supporting methods and tables for hedonic model for property sales value.**
(DOC)
