# Correction: How Much Does it Cost to Expand a Protected Area System? Some Critical Determining Factors and Ranges of Costs for Queensland

**DOI:** 10.1371/annotation/27546408-8e16-4998-8886-28787d1772f9

**Published:** 2011-11-08

**Authors:** Vanessa M. Adams, Daniel B. Segan, Robert L. Pressey

The Funding line should read: "The authors acknowledge funding for this research from WWF-Australia. RLP acknowledges support from the Australian Research Council. The funders had no role in study design, data collection and analysis, decision to publish, or preparation of the manuscript." 

