# Supplementary material for: How Much Does it Cost to Expand a Protected Area System? Some Critical Determining Factors and Ranges of Costs for Queensland
Source: PLoS One. 2011 Sep 28;6(9):e25447. doi: 10.1371/journal.pone.0025447 (PMC3182235; doi:10.1371/journal.pone.0025447)
Supplement: Information S1 — Supporting methods and tables for hedonic model for property sales value. (DOC) [file pone.0025447.s001.doc]

**Supporting Information S1**

Hedonic model for property sales value

The market value of a property can be estimated as the sale value in an open land market. We estimated market values from recent sales of properties in Queensland from 2000-2008 [1,2,3]. Dates of land valuation and sales data varied, so we adjusted all values to 2008 dollars using published annual inflation rates [4]. The land value, sale value, tenure and size of each property were recorded from the sales valuation data. In addition, we sourced property characteristics from state-wide data including the area on each property covered by soil of high productivity, the area on each property that was cleared, distance to nearest town, and occurrence in coastal regions (Table S1). Other potential explanatory variables, such as current land use, elevation and slope were not consistently mapped for the entire state. We specified a hedonic price model for the market value with the dependent variable being the log of the sale value per hectare. The hedonic analysis was modelled using two methods: 1. ordinary least squares (OLS) estimation in R [5] and 2. Geographically weighted regression in ArcGIS 9.3.

Full models can result in over-fitting, particularly when the model is used to predict values [6]. Therefore, for the hedonic model using OLS, a reduced model was selected using full forward and backward stepwise removal using the Akaike information criterion (AIC) [7] to select the best model using the ‘step’ function in R [5] (Table S2).

The sale value per hectare was significantly higher in South East Queensland than all other regions of the state. The sale value per hectare for other coastal areas was also significantly higher than non-coastal properties. Cape York had low sale values per hectare. This is in line with expectations of the land market. Brisbane and surrounding coastal areas in the South East portion of the state have significantly higher sale values while Cape York Peninsula is predominantly state and aboriginal land with only a small portion of freehold land being sold around town sites such as Weipa. For coastal regions, sale value decreased with increasing distance to towns. For non-coastal regions the coefficient of *log(distance to nearest town, km)* was positive indicating that sale value increased with increasing distance to towns. This likely reflects the fact that inland leasehold properties with high sale values are often located far from townships. The three variables used to capture existing and potential development were *log(cleared area, ha)*, *log(kandasol soil, ha)* and *log(vertosol soil, ha)*. These were all positively related to sale value.

For the geographically weighted regression we divided the properties in Queensland into coastal and non-coastal properties due to the large variation in characteristics such as size (for example average size for coastal properties is 1/40 the size of properties across the remainder of the state). For coastal properties the only predictor without strong local correlations was *log(cleared area, ha)* and adjusted R^2^  was 0.688. For non-coastal properties the final variables included were *log(land value per ha)*, *log(cleared area, ha)*, *log(soil, ha)* and *log(distance to nearest town, km)* (adjusted R^2^=0.904). The values predicted by the geographically weighted regression were highly correlated with the OLS predicted values, suggesting that our regional dummy variables captured some of the local spatial drivers.

**Table S1** Predictors considered and sources of data

| Predictor | Description | Source |
| --- | --- | --- |
| log(property area, ha) | Log of area of property in ha | [2] |
| log(land value per ha) | Log of land value per ha | [1] |
| log(cleared area, ha) | Log of cleared area of property in ha as a surrogate measure of existing development | [8] |
| log(kandasol soil, ha) | Log of area of property in ha with kandasol soils as a surrogate measure of suitability for agricultural development | [9] |
| log(vertosol soil, ha) | Log of area of property in ha with vertosol soils as a surrogate measure of suitability for agricultural development | [9] |
| log(soil, ha) | Log of area of property in ha with vertosol or kandasol soils as a surrogate measure of suitability for agricultural development | [9] |
| Tenure: Leasehold (LH), Freehold (FH) | Dummy variable: Leasehold (LH) | [2] |
| log(distance to nearest town, km) | Log of distance to nearest town in km as a surrogate measure of proximity to urban services | [10] |
| Regions: South East Queensland, Coastal Queensland, Cape York Peninsula, Remainder of State | Dummy variables: South East Queensland (all coastal local government areas from the southern border to the Sunshine Coast), Cape York (Cape York Peninsula) and Coastal Queensland (all other Coastal local government areas) | [11] |
| LHxlog(property area, ha) | Interaction term for tenure and property size |  |
| SExlog(distance to nearest town, km) | Interaction term for South East Queensland and distance to town |  |
| Coastalxlog(distance to nearest town, km) | Interaction term for Coastal Queensland and distance to town |  |
| CapeYorkxlog(distance to nearest town, km) | Interaction term for Cape York and distance to town |  |

**Table 2 Hedonic coefficient estimates for sale value in 2008 using the reduced model with OLS**

| Variable | Coefficient | Std. Error | Pr(>t) |
| --- | --- | --- | --- |
| log(land value per ha) | 0.5035 | 0.00457 | 0.0001 |
| log(property area, ha) | -0.4876 | 0.00524 | 0.0001 |
| log(cleared area, ha) | 0.60291 | 0.00170 | 0.0001 |
| log(kandasol soil, ha) | 0.0193 | 0.00472 | 0.0001 |
| log(vertosol soil, ha) | 0.0893 | 0.00482 | 0.0001 |
| log(distance to nearest town, km) | 0.0358 | 0.01012 | 0.0001 |
| South East Queensland | 0.2197 | 0.01455 | 0.0001 |
| Cape York | -0.1812 | 0.02794 | 0.0001 |
| Coastal | 0.1579 | 0.01543 | 0.0001 |
| LHxlog(property area, ha) | 0.0662 | 0.00549 | 0.0001 |
| SExlog(distance to nearest town, km) | -0.1792 | 0.01242 | 0.0001 |
| Coastalxlog(distance to nearest town, km) | -0.1600 | 0.01308 | 0.0001 |
| Constant | 2.818 | 0.02388 | 0.0001 |
| N=41901 parcels |  |  |  |
| R^2^ (adjusted) =0.901 |  |  |  |

**References**
